# Uncertainty of Liver Cirrhosis Diagnosis and Use of Elastography

**DOI:** 10.7759/cureus.18411

**Published:** 2021-09-30

**Authors:** Daniel M Aloise, Guillermo Izquierdo

**Affiliations:** 1 Translational Medicine, Herbert Wertheim College of Medicine, Florida International University, Miami, USA; 2 Internal Medicine, Herbert Wertheim College of Medicine, Florida International University, Miami, USA

**Keywords:** cirrhosis diagnosis, maddrey's discriminant function, meld score, elastography, alcoholic cirrhosis, liver cirrhosis, severe alcoholic hepatitis, jaundice

## Abstract

A case of severe jaundice in a patient with a long history of alcohol abuse led to a questionable diagnosis of liver cirrhosis. To determine its diagnostic utility in the setting of liver disease, elastography was utilized on our patient to confirm the clinically suspected diagnosis of cirrhosis.

A 59-year-old male presented to our emergency department (ED) with two days of progressive jaundice and right upper quadrant (RUQ) pain. The patient admitted to drinking > 500 mL of vodka daily for the last seven years, with his last drink on the morning of admission. Physical exam revealed a man in mild acute distress with severe jaundice and an abdomen diffusely tender to palpation. Two spider angiomas were present on the torso along with caput medusae and mild asterixis. Labs revealed aspartate aminotransferase (AST) 408, alanine aminotransferase (ALT) 69, prothrombin time (PT) 16.3, partial thromboplastin time (PTT) 36, total bilirubin 22.6, and direct bilirubin 19.9 mg/dL. While admitted, total bilirubin rose as high as 31.5 mg/dL. Examination showed a Model for End-Stage Liver Disease (MELD) score of 22 and a Maddrey score of 37. Ultrasound revealed moderate hepatosplenomegaly with no signs of pancreatitis.

Based on the patient’s history of alcohol abuse paired with physical exam findings and elevated laboratory markers, we were able to diagnose with a high level of suspicion that this patient was suffering from chronic alcoholic liver disease, exacerbated by an acute episode of alcoholic hepatitis, which led to hepatic encephalopathy. Based on these findings, a diagnosis of liver cirrhosis was suspected; however, this diagnosis required further confirmation. We utilized ultrasound elastography to measure the velocity of shear wave transmission in the liver of our patient. A literature review was conducted on the use of elastography for the diagnosis of liver disease, and a significant correlation between the velocity of shear wave transmission and hepatic histological findings was identified. Elastography revealed a mean velocity of shear wave transmission of 1.77 m/s in our patient. This finding is consistent with a Meta-analysis of Histological Data in Viral Hepatitis (METAVIR) score of F = 4, indicating significant fibrosis and confirming the suspected diagnosis of alcohol-induced liver cirrhosis.

As a non-invasive and inexpensive diagnostic tool, elastography demonstrates significant potential for clinical utility in patients with liver disease. Clinicians may benefit from the use of elastography in diagnosis, while patients may receive both therapeutic and prognostic benefits secondary to its use. In similar cases with clinical uncertainty, elastography can reliably identify the presence of fibrous tissue in the liver without tissue biopsy, thus aiding in clinical diagnoses and enabling the use of optimal therapeutic regimens for future patients.

## Introduction

We propose the use of non-invasive, inexpensive elastography to identify liver cirrhosis in patients with suspected, yet uncertain, clinical diagnoses of cirrhosis. A widely available yet underutilized diagnostic tool, elastography, demonstrates the potential for significant clinical utility in patients with liver disease [1,2]. Clinicians may benefit from the use of elastography as a diagnostic tool, while patients may receive both therapeutic and prognostic benefits secondary to its use [3]. Elastography was used in a questionable case to determine the presence of cirrhosis.

## Case presentation

A 59-year-old male with a history of alcohol abuse presented to our emergency department due to two days of progressive jaundice and right upper quadrant (RUQ) pain. The patient admitted to drinking at least 500 mL of vodka (> 100g alcohol) daily for the last seven years with his last drink on the morning of admission. He also reported nausea with decreased appetite, dark urine, and dark stools, but denied vomiting and diarrhea. The patient’s most recent hospitalization was six months ago for altered mental status secondary to alcohol use. Physical exam revealed a man in mild acute distress with severe jaundice affecting the sclera of the eyes, torso, thighs, hands, and feet, along with an abdomen diffusely tender to palpation. Mild ascites was noted along with mild hepatosplenomegaly. Two spider angiomas were present on the torso along with caput medusae and mild asterixis. Labs revealed aspartate aminotransferase (AST) 408, alanine aminotransferase (ALT) 69, prothrombin time (PT) 16.3, partial thromboplastin time (PTT) 36, total bilirubin 22.6, and direct bilirubin 19.9 mg/dL. While admitted, total bilirubin rose as high as 31.5 mg/dL. Ultrasound showed moderate hepatosplenomegaly with increased echogenicity of the liver and no signs of pancreatitis.

Based on the patient’s reported history of alcohol abuse paired with physical exam findings (jaundice, RUQ pain, spider angiomas, caput medusae, and asterixis) and elevated laboratory markers, we were able to diagnose with a high level of suspicion that this patient was suffering from chronic alcoholic liver disease, exacerbated by an acute episode of alcoholic hepatitis, which led to hepatic encephalopathy. Based on the aforementioned findings, a diagnosis of cirrhosis of the liver was suspected; however, this diagnosis required further confirmation.

We utilized ultrasound elastography to verify our suspected clinical diagnosis of liver cirrhosis. Elastography utilizes shear wave transmission technology to estimate the elasticity of tissue [2]. Stiffer tissue transmits waves faster than softer, more flexible tissue. Thus, the velocity with which these waves travel through the liver can indicate histological changes that occur with chronic alcohol use. When native hepatocytes are replaced by stiffer, more fibrotic tissue, this tissue will conduct waves at a faster velocity [2]. Our patient displayed a mean velocity of shear wave transmission of 1.77 m/s with a standard deviation of 0.28 m/s. These findings are consistent with a Meta-analysis of Histological Data in Viral Hepatitis (METAVIR) score of F = 4, indicating significant fibrosis corresponding with liver cirrhosis [4]. A CT scan of the abdomen revealed no evidence of pancreatitis. The patient exhibited an elevated Model for End-Stage Liver Disease (MELD) score of 22 and elevated Maddrey’s discriminant function score of 38, consistent with severe alcoholic hepatitis with a poor prognosis [5]. According to an article by Crabb et al. in 2016, a MELD score > 20 predicts 90-day mortality of 20%, while patients with a Maddrey's discriminant function score > 31 have a one-month mortality rate as high as 20-50% [5]. The patient was treated with a symptomatic approach for encephalopathy without steroids due to possible concomitant pancreatitis and/or infection. We provided lactulose and enteral nutrition along with empiric antibiotics. Together, this led to clinical and symptomatic improvement in the patient.

**Figure 1:**
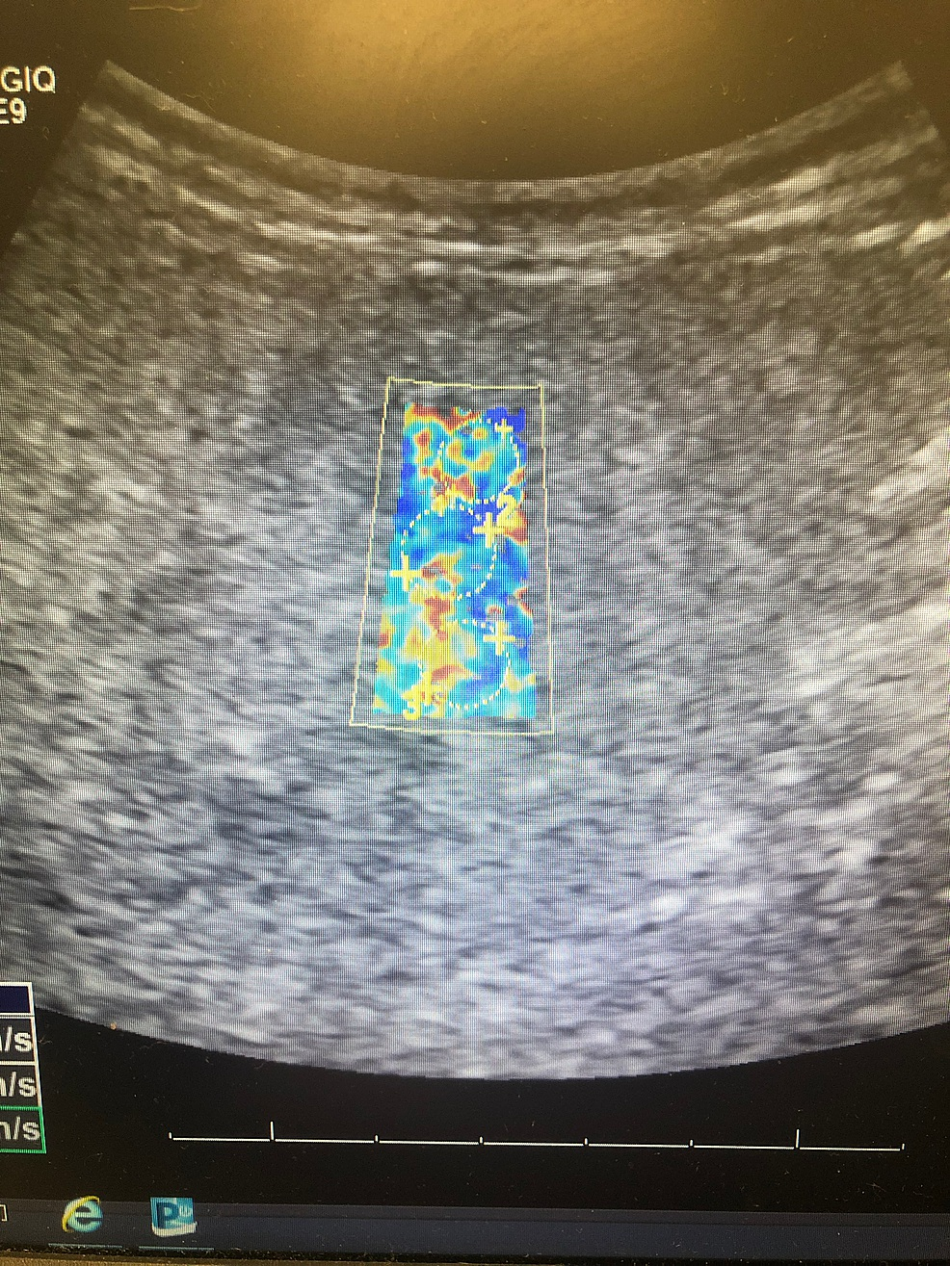
Elastography of Patient's Liver The first three of twelve separate sections of our patient's liver examined using elastography. Note heterogenous blue, green, yellow, orange, and red admixed, indicating increasing areas of fibrosis.

Included are images of elastography taken of our patient’s liver, courtesy of Vicente Hernandez from the Jackson Memorial Hospital Ultrasound Department. Twelve regions are selected from a patient’s liver to calculate a mean velocity of transmission, indicating the degree of elasticity, which correlates with underlying tissue fibrosis. Figure 1 shows an image of our patient’s liver, which displays significant areas of heterogeneous yellow and red intermixed with blue, the former of which correspond with areas of increasing elasticity.

**Figure 2:**
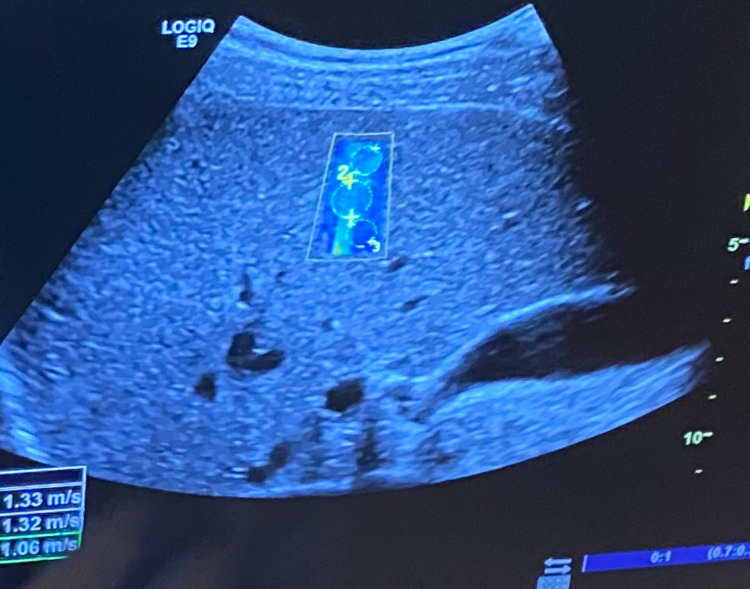
Elastography of Medical Student's Liver Homogenously blue liver, indicating the normal velocity of shear wave transmission (1.33 m/s). Elastography indicates normal hepatocytes with no sign of fibrosis (METAVIR F = 0).

Figure 2 shows an image of elastography conducted on the liver of a 25-year-old medical student, Daniel Aloise, representing a comparison to a healthy, non-cirrhotic liver. Note that the image is homogenously blue throughout, with no signs of increased elasticity or fibrosis. The liver in Figure 2 transmitted shear waves at an average velocity of 1.33 m/s, corresponding with a METAVIR score of F = 0. This is compared to 1.77 m/s as the mean velocity of shear wave transmission in our patient's liver tissue, corresponding with a METAVIR score of F = 4.

**Figure 3:**
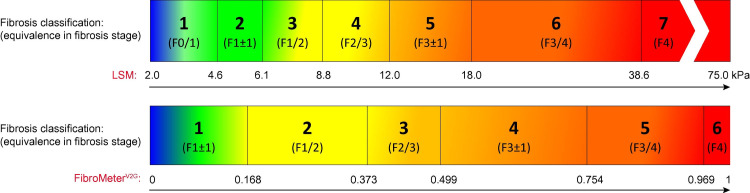
Elastography Color with METAVIR Score LSM = Liver Stiffness Measurement kPa = kilo Pascal, measurement of liver stiffness METAVIR = Meta-analysis of Histological Data in Viral Hepatitis The figure depicts approximate color seen in Ultrasound with the corresponding degree of liver elasticity/fibrosis (METAVIR score F = 0-4)

Figure 3 shows the approximate color correlation with degree of fibrosis, according to a study conducted by Boursier et al. in 2016 [6].

Table 1 depicts the reference values for METAVIR scoring of liver fibrosis, according to a study conducted by Osman et al. in 2020 [4]. Reference METAVIR values for liver fibrosis are as follows: F0 for velocities < 1.47 m/s. F1: for velocities 1.47-1.48 m/s. F2: for velocities 1.48 - 1.64 m/s. F3: for velocities 1.64 - 1.76 m/s. F4: for velocities > 1.76 m/s [4]. 

**Table 1:** Liver Fibrosis Staging and METAVIR Score based on Elastography Velocity m/s = meters per second METAVIR = Meta-analysis of Histological Data in Viral Hepatitis

Liver Fibrosis Staging	METAVIR Score	m/s
Normal	F0	< 1.47
Normal-mild	F1	1.47 – 1.48
Mild-moderate	F2	1.48 – 1.64
Moderate-severe	F3	1.64 – 1.76
Cirrhosis	F4	> 1.76

**Figure 4:**
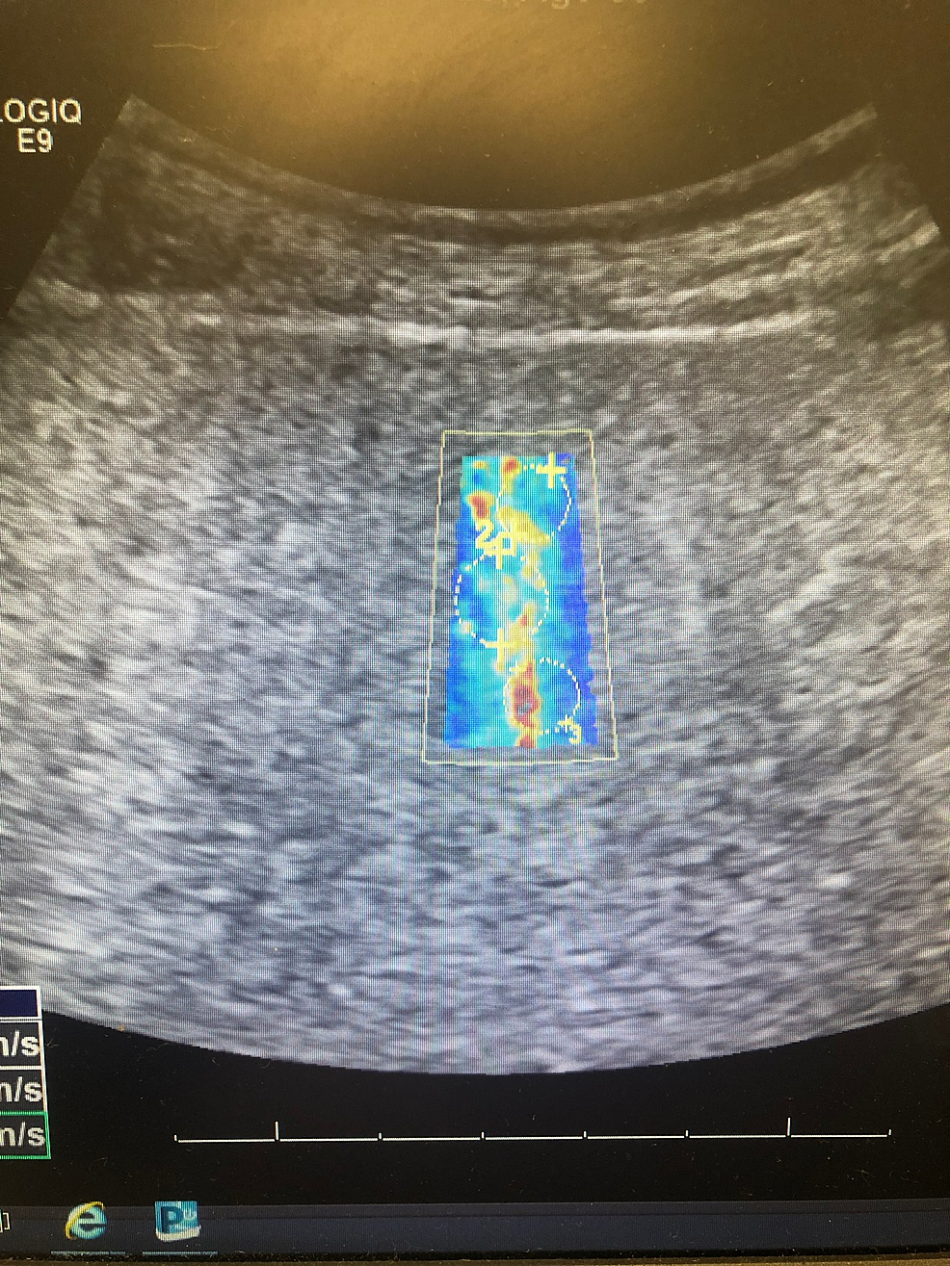
Elastography of Patient's Liver #2 The second set of sections from our patient's liver examined using elastography. Each circle indicates a section's velocity measured within the highlighted region. Heterogenous blue, green, yellow, orange, and red admixed indicate increased shear wave velocity, corresponding with increasing underlying fibrotic histology.

**Figure 5:**
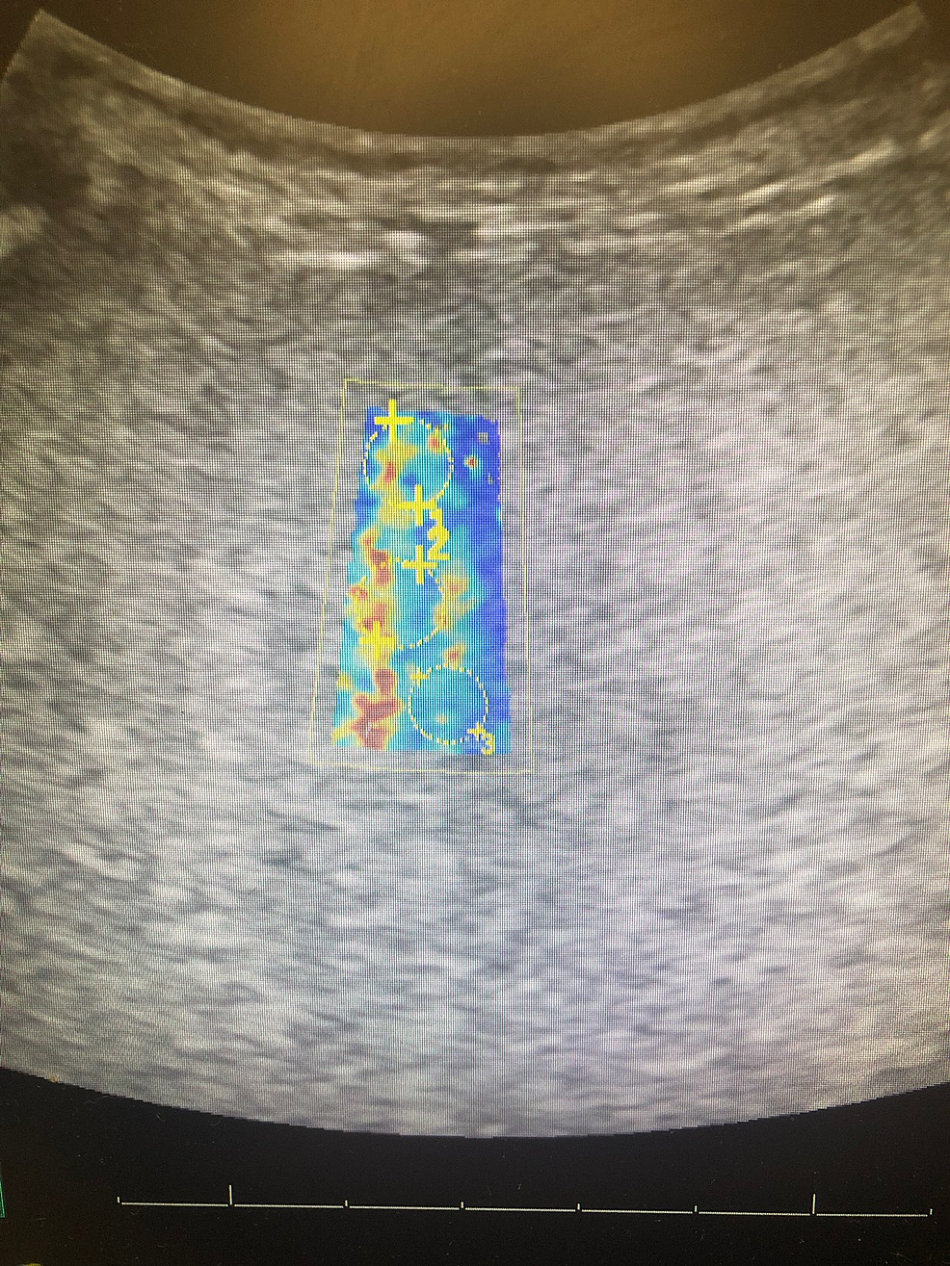
Elastography of Patient's Liver #3 The third set of sections examined from our patient's liver using elastography. Three circles are shown in each of the four highlighted images included for a total of 12 sections, with the velocity of shear wave transmission measured in each.

**Figure 6:**
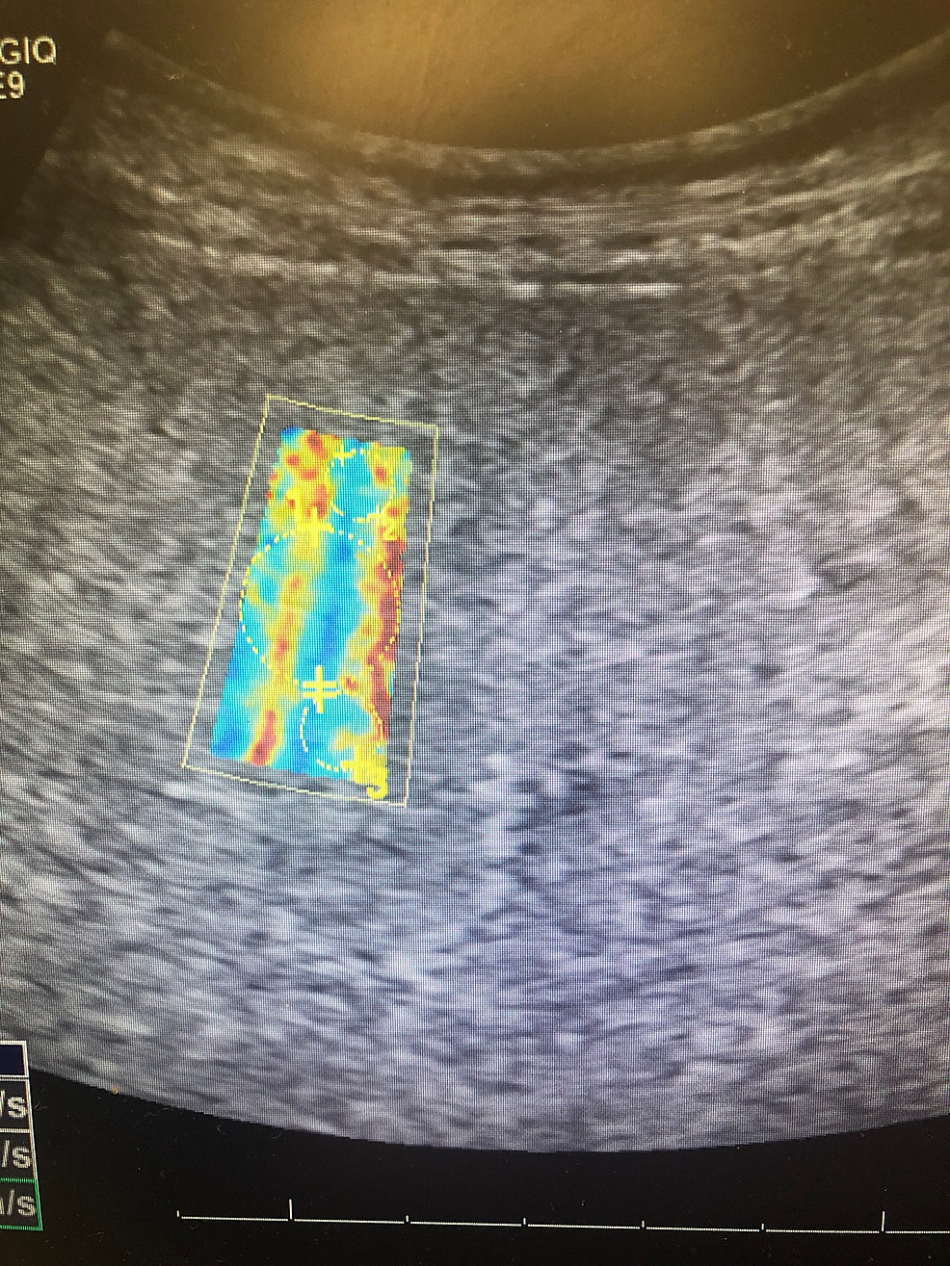
Elastography of Patient's Liver #4 The fourth and final set of sections measured from our patient's liver using elastography. The mean velocity was calculated from these 12 sections to calculate a mean transmission velocity of 1.77 m/s, which correlates with a Meta-analysis of Histological Data in Viral Hepatitis  (METAVIR) score of F = 4, consistent with liver cirrhosis.

Figures 4-6 show additional images depicting the twelve regions utilized for elastography in our patient’s liver.

 

## Discussion

Elastography estimates liver stiffness by applying mechanical waves and measuring their propagation speed through the tissue with the use of imaging [2]. Modality options include ultrasound (i.e., transient elastography, acoustic radiation force impulse imaging, two-dimensional shear wave elastography) and MRI [2]. The general principle behind the use of this technology is simple: mechanical waves, specifically shear waves, travel faster through stiffer tissue than through softer tissue. As such, the faster these shear waves travel through a patient’s liver, the stiffer the tissue is, thus correlating with the degree of fibrosis, and ultimately, cirrhosis. Elastography can provide clinicians with a METAVIR score ranging from 0 to 4, ranging from no fibrosis (F= 0) to significant fibrosis (F = 2), to cirrhosis (F = 4) [4].

Liver cirrhosis is a diagnosis with severe consequences, as the replacement of native hepatocytes with nonfunctional fibrotic tissue is often irreversible, necessitating liver transplant for long-term patient survival [7]. Cirrhosis can lead to a multitude of severe and potentially lethal complications, including variceal hemorrhage, hepatorenal syndrome, hepatopulmonary syndrome, hepatic encephalopathy, cirrhotic cardiomyopathy, and hepatocellular carcinoma [7]. Cirrhosis is a diffuse process of liver damage that is irreversible in advanced stages, yet with treatment goals, lifestyle modifications, and recommended screenings we can improve the poor prognosis of many patients diagnosed with cirrhosis [3]. In 2016, more than 40,000 Americans died from complications related to cirrhosis, making cirrhosis the 12th leading cause of death in the United States [3]. Projections estimate that this number will rise, as the incidence of nonalcoholic steatosis continues to increase in the US, disproportionately affecting non-Hispanic blacks, Mexican-Americans, and those living below the poverty level [3]. There are an estimated 630,000 Americans with cirrhosis, yet less than one in three knows it [3]. Cirrhosis and advanced liver disease kill tens of thousands of Americans each year and cost the United States between $12 billion and $23 billion dollars in health care expenses annually [3].

The gold standard for diagnosis of cirrhosis is liver biopsy, though this is an invasive process that requires several biopsies from multiple lobes of the liver [2]. Many serologic and imaging tests have been proposed as tools to diagnose cirrhosis, though none have emerged as the universal standard of care for cirrhosis diagnosis. Elastography has been identified as a viable diagnostic tool that can reliably identify fibrous tissue in the liver, corroborating a clinically suspected diagnosis of liver cirrhosis [2]. Studies have shown the efficacy of elastography to be clinically significant.

A study performed by Friedrich-Rust et al. in 2006 showed a highly significant (p < 0.001) Spearman’s correlation coefficient of 0.48 between elasticity scores obtained using real-time elastography and histologic fibrosis stage [8]. The diagnostic accuracy expressed as areas under receiver operating characteristic (ROC) curves were 0.75 for the diagnosis of significant fibrosis (fibrosis stage according to METAVIR scoring system [F] ≥ F2), 0.73 for severe fibrosis (F ≥ F3), and 0.69 for cirrhosis. For a combined elasticity-laboratory score, the areas under the ROC curves were 0.93, 0.95, and 0.91, respectively [8]. Another study by Berends et al. in 2007 showed that in a study of 24 patients who were taking methotrexate and had undergone a liver biopsy, elastography correctly identified 88% of patients who did not have significant fibrosis, while FibroTest identified 83% of patients with significant fibrosis [9]. FibroTest is an identical European proprietary test to FibroSure in America, that involves assessment of alpha-2-macroglobulin, alpha-2-globulin (haptoglobin), gamma globulin, apolipoprotein A1 (ApoA1), gamma-glutamyl transferase (GGT), and total bilirubin, while also taking into account the patient's age and sex [10]. Results from the individual assays are combined and used to classify patients having mild fibrosis (F0 to F1), significant fibrosis (F2 to F4), or an indeterminate stage of fibrosis. The sensitivity for detection of significant fibrosis is approximately 60 to 75 and the specificity is approximately 80 to 90 percent [11-14]. In a study performed by Castera et al. in 2005 consisting of 183 patients with chronic hepatitis C virus (HCV) infection, the combination of elastography with FibroTest demonstrated an area under the ROC curve of 0.88 for F ≥2, 0.95 for F ≥3, and 0.95 for F = 4 [15]. When the elastography and FibroTest results agreed, liver biopsy examination confirmed the stage of fibrosis in 84% of cases for F ≥2 fibrosis, in 95% for F ≥3 fibrosis, and in 94% for F = 4 fibrosis [15]. A systematic review conducted by Geng et al. in 2016 incorporated a meta-analysis on 57 studies, which included 10,504 patients, of which 1,870 had cirrhosis, calculating the sensitivity and specificity of elastography in diagnosing liver cirrhosis [1]. This was in comparison to the gold standard of liver biopsy. The pooled sensitivity of transient elastography in assessing liver cirrhosis was calculated to be 81% (95% CI: 79%-83%) [1]. The pooled specificity of transient elastography in assessing liver cirrhosis was found to be 88% (95% CI: 87%-89%) [1]. Based on these findings, it is likely that a combination of serum biomarkers paired with elastography will improve the accuracy of fibrosis detection while preventing the necessity of more invasive and expensive diagnostic tools [3].

The diagnosis of liver cirrhosis is an important one in terms of patient prognosis. The overall 30-day mortality rate in patients hospitalized with alcoholic hepatitis is about 15%; however, in patients with severe liver disease, this rate approaches or exceeds 50% [16]. Overall, the one-year mortality rate following hospitalization for alcoholic hepatitis is about 40% [16]. Annualized rates of progression of the pre-cirrhotic disease to liver cirrhosis are reported to be 1% (0-8%) for patients with normal histology, 3% (2-4%) for hepatic steatosis, 10% (6-17%) for steatohepatitis, and 8% (3-19%) for liver fibrosis. [16]. By enabling physicians to effectively diagnose liver cirrhosis in an inexpensive and non-invasive manner, elastography provides the means for clinicians to more accurately define the prognosis of their patients and treat them accordingly. This case report serves as a prime example of a patient that stood to benefit from the use of elastography. Based on its diagnostic effectiveness in addition to its relative cost and non-invasive manner, elastography shows promise as a clinical tool that may be considered for incorporation into the prognosis scores for patients suffering from chronic liver disease [1]. This case report may generate additional research on the diagnostic utility of elastography. We recommend future studies to investigate further, so we may ultimately consider changes in the current standard of care for patients suffering from liver disease.

## Conclusions

Elastography shows promise as an efficacious diagnostic tool for liver disease. In contrast with the current gold standard for diagnosis of cirrhosis, which involves several invasive liver biopsies from multiple lobes of the liver, elastography offers a non-invasive approach to the diagnosis of this increasingly prevalent disease. Studies have shown elastography to be effective, especially when these results are paired with serum biomarkers and clinical findings for diagnosis. By providing a readily available ultrasound option, we can effectively diagnose cirrhosis in an inexpensive, patient-friendly, and efficient manner. Elastography may enable clinicians to accurately diagnose more patients with cirrhosis, as the majority of patients are not even aware of their underlying disease, likely due in part to the invasive nature of the previous gold standard for diagnosis and lack of a consensus secondary diagnostic tool. By efficiently diagnosing or excluding cirrhosis, healthcare workers can provide their patients with the optimal therapeutic care and appropriate screenings that align with their respective diagnostic findings. In doing so, clinicians may ultimately improve patient prognosis, thus exhibiting both the therapeutic and prognostic utility of elastography.
